# The Role of Headwater Streams in Downstream Water Quality^1^

**DOI:** 10.1111/j.1752-1688.2007.00005.x

**Published:** 2007-02

**Authors:** Richard B Alexander, Elizabeth W Boyer, Richard A Smith, Gregory E Schwarz, Richard B Moore

**Keywords:** rivers/streams, nitrogen, transport and fate, streamflow, headwaters, *SWANCC*, Rapanos

## Abstract

Knowledge of headwater influences on the water-quality and flow conditions of downstream waters is essential to water-resource management at all governmental levels; this includes recent court decisions on the jurisdiction of the Federal Clean Water Act (CWA) over upland areas that contribute to larger downstream water bodies. We review current watershed research and use a water-quality model to investigate headwater influences on downstream receiving waters. Our evaluations demonstrate the intrinsic connections of headwaters to landscape processes and downstream waters through their influence on the supply, transport, and fate of water and solutes in watersheds. Hydrological processes in headwater catchments control the recharge of subsurface water stores, flow paths, and residence times of water throughout landscapes. The dynamic coupling of hydrological and biogeochemical processes in upland streams further controls the chemical form, timing, and longitudinal distances of solute transport to downstream waters. We apply the spatially explicit, mass-balance watershed model SPARROW to consider transport and transformations of water and nutrients throughout stream networks in the northeastern United States. We simulate fluxes of nitrogen, a primary nutrient that is a water-quality concern for acidification of streams and lakes and eutrophication of coastal waters, and refine the model structure to include literature observations of nitrogen removal in streams and lakes. We quantify nitrogen transport from headwaters to downstream navigable waters, where headwaters are defined within the model as first-order, perennial streams that include flow and nitrogen contributions from smaller, intermittent and ephemeral streams. We find that first-order headwaters contribute approximately 70% of the mean-annual water volume and 65% of the nitrogen flux in second-order streams. Their contributions to mean water volume and nitrogen flux decline only marginally to about 55% and 40% in fourth- and higher-order rivers that include navigable waters and their tributaries. These results underscore the profound influence that headwater areas have on shaping downstream water quantity and water quality. The results have relevance to water-resource management and regulatory decisions and potentially broaden understanding of the spatial extent of Federal CWA jurisdiction in U.S. waters.

## Introduction

Recent U.S. Supreme Court rulings, related to Clean Water Act (CWA) decisions by federal regulatory agencies (U.S. Army Corps of Engineers and U.S. Environmental Protection Agency), underscore the need for an improved scientific understanding of the influence of headwater areas and upland (low-order) streams on the physical, chemical, and biological integrity of downstream waters, especially those legally classified as “navigable.” An important 2001 U.S. Supreme Court ruling (*Solid Waste Agency of Northern Cook County v. U.S. Army Corps of Engineers*; *SWANCC*) and subsequent court decisions interpreting the meaning of *SWANCC* focused on the scope of the CWA permit program as it applies to land development, and have raised questions about the jurisdiction of federal regulatory agencies over various U.S. waterways. The *SWANCC* case narrowed federal authority to protect many upstream and wetland areas, stated as isolated, non-navigable, intrastate waters that are not tributary or adjacent to navigable waters or their tributaries. In subsequent appellate circuit decisions, many questions have been raised about how to interpret the *SWANCC* decision (e.g., the definition of “adjacent”) and about what parts of the tributary system are considered jurisdictional under the CWA. These decisions include several recent cases (2006: *Rapanos v. United States*, 04-1034, *Carabell v. Army Corps of Engineers*, 04-1384, and S.D. Warren Co. *v.* ME Board of Environmental Protection, 04-1527) that have not resolved questions about which wetland areas are protected by the CWA.

An improved scientific understanding of the influence of headwater streams on the integrity of downstream navigable waters (especially those that may have less obvious relationships to navigable-in-fact waters; see Federal Register, 2003) is viewed as a central need to assist policy makers, regulatory authorities, and the courts. Of particular interest in determining CWA jurisdiction is whether a “significant nexus” exists between upstream waters and navigable-in-fact waters. Such a connection could be based on evidence that the use, degradation, or destruction of non-navigable headwaters demonstrably affects downstream navigable waters and their tributaries. However, legal ambiguities currently exist as to what constitutes “navigable streams and their tributaries”– i.e., how far upstream does CWA jurisdiction actually extend into tributary reaches. A recent 2006 U.S. Supreme Court decision on the consolidated cases of *Rapanos v. United States & Carabell v. Army Corps of Engineers* failed to explicitly resolve these questions. The ruling specified that Federal CWA jurisdiction requires evidence of a “significant nexus” between upstream waters and navigable waters, based on a technical and scientific judgment by Federal regulators. The cases were remanded to the lower courts for re-evaluation under these guidelines.

Our study provides scientific insight into the coupled hydrological, chemical, and biological influences of headwater systems on downstream navigable waters and their tributaries. An earlier synthesis effort (Nadeau and Leibowitz, 2003) summarized current scientific knowledge of the hydrological and biologic connections between “isolated” wetlands and downgradient surface-water systems. Although a broad range of types of material fluxes and concentrations in headwater and larger streams is ultimately of interest in discussions of headwater connectivity, we focus in this study exclusively on a discussion of nitrogen fluxes in surface waters.

Nitrogen is an essential nutrient that regulates primary production in terrestrial and aquatic ecosystems. Nitrogen inputs to landscapes have increased markedly over the past 50 years across the globe in response to increased food and energy production, which has created an abundant supply of highly reactive forms of nitrogen in air, land, and water (Galloway *et al.*, 2004). Excess nitrogen has been linked to many environmental concerns, including the disruption of forest ecosystem processes (Aber *et al.*, 2003), acidification of lakes and streams (Driscoll *et al.*, 2001), and degradation of coastal waters including high profile water quality issues such as eutrophication, hypoxia, and harmful algal blooms (NRC, 2000). Nitrogen is also the focus of recent USEPA efforts to establish nutrient criteria in U.S. streams, lakes, and estuaries (USEPA, 2000). Moreover, because nitrogen is highly reactive and mobile in terrestrial and aquatic ecosystems, it also serves as a relatively suitable surrogate for many contaminants and potentially toxic substances in water where understanding of the linkages between headwaters and downstream receiving waters is important. Although the complexities of nitrogen cycling in terrestrial and aquatic ecosystems are notable, a considerable body of experimental research and large-scale budgeting and modeling analyses has emerged to support reliable descriptions of the sources and transport of nitrogen over broad spatial scales within streams and rivers.

Our study is organized in two major sections. The first section provides an overview of the principal conceptual frameworks and current watershed research relevant to evaluating the role of headwater streams in controlling nitrogen conditions in downstream waters. This synthesis illustrates current understanding of the coupling of land use, pollutant sources, and hydrological and biogeochemical processes on the landscape and how these activities and processes control the supply and delivery of water and nitrogen flux to headwater streams. We further examine the function that stream channels play in controlling water routing and instream processing and their effects on nitrogen transport from headwaters to downstream waters.

In the second section of the article, we use the water-quality model SPARROW (SPAtially Referenced Regression On Watershed attributes; Smith *et al.*, 1997) to investigate and quantify headwater influences in streams of the northeastern United States. SPARROW is a hybrid statistical/mechanistic watershed model with mass-balance constraints. The model descriptions of landscape and aquatic processes are sufficiently detailed to support an assessment of the effects of headwater processes and pollutant sources on water-quality conditions throughout large river networks. Although progress has been made in empirically modeling the transport of nitrogen in streams (e.g., Seitzinger *et al.*, 2002), most empirical watershed models lack mass-balance constraints and do not separate land and water processes. These features are necessary to accurately quantify nutrient transport in streams of varying sizes in river networks (e.g., Smith *et al.*, 1997; Alexander *et al.*, 2002a,b;). Moreover, dynamic mechanistic watershed models (e.g., HSPF; Bicknell *et al.*, 2001), although providing detailed predictions of nitrogen flux over time in response to short-term changes in climate, hydrology, and nutrient cycling dynamics, are frequently applied only in small catchments and lack the spatial detail and observational data needed to quantify the fate of headwater nitrogen sources and cycled nitrogen in large river networks. To enhance our model-based descriptions of nitrogen transport from headwaters to downstream navigable waters and their tributaries, we modify the structure of a previous SPARROW model (Moore *et al.*, 2004) to incorporate observations of nitrogen removal in streams and lakes from the primary literature. We use the refined model to assess the effects of streamflow and nitrogen supply and removal processes in headwaters on the flow and nitrogen conditions in downstream waters.

## The complex interactions of nitrogen in watersheds

### Landscape and Water Interactions

Although nutrients are associated with healthy watersheds and the provision of ecosystem services, they also can act as pollutants. Commonly described as “too much of a good thing,” it is the overabundance of nitrogen loadings that leads to negative environmental effects. Nitrogen in the environment has vastly increased in recent decades, largely associated with growing populations and associated land use, from: (1) creation of reactive nitrogen, via the Haber-Bosch process, for fertilizers and other industrial applications; (2) cultivation of vast land areas of crops that host nitrogen-fixing bacteria; and (3) fossil fuel burning and the associated emissions and nitrogen deposition (Smil, 2001). Worldwide, human activities have more than doubled the amount of reactive N entering the environment (Vitousek *et al.*, 1997; Galloway *et al.*, 2004). In an individual watershed, the distribution of human and animal populations, land use, and characteristics of the vegetation and soils set the stage for the types, magnitudes, and geography of nitrogen inputs (Boyer *et al.*, 2002).

Stemming from nitrogen inputs to landscapes, nitrogen fluxes in many surface waters have increased in recent decades, and two-thirds of the nation’s estuaries are degraded from nitrogen pollution (Bricker *et al.*, 1999). Nitrogen flux in streams and rivers of any size is the cumulative result of processes that control the supply and transport of nitrogen in terrestrial and aquatic ecosystems. These occur throughout the watershed system from the headwater source areas to the downstream receiving waters (Howarth *et al.*, 1996; Seitzinger *et al.*, 2002; Van Breemen *et al.*, 2002; McClain *et al.*, 2003). As a result, nitrogen pollution and other nutrient problems are increasingly being addressed by researchers and management agencies by considering the intrinsic linkages between terrestrial upland landscapes and the aquatic systems to which they drain (Driscoll *et al.*, 2003; Grimm *et al.*, 2003).

Nitrogen fluxes in surface waters are controlled to a large degree by heterogeneous distributions of nitrogen inputs (Howarth *et al.*, 1996; Boyer *et al.*, 2002). The environmental setting – e.g., climate, topography, vegetation, and soil properties – also shapes both land use (and the types of nitrogen sources) and how nitrogen inputs are mediated. Nitrogen is highly reactive, ensuring biogeochemical processing and transformations in landscapes, including nutrient production mechanisms, assimilation and uptake in plant material, and permanent removal via denitrification (Davidson and Schimel, 1995; Van Breemen *et al.*, 2002; Boyer *et al.*, 2006b). Denitrification is a process whereby the reactive forms of nitrogen are transformed into dinitrogen (N_2_) gas, which is highly inert and does not have any adverse environmental consequences (and, in fact, is the dominant component of the earth’s atmosphere). Further, nitrogen is highly soluble and is transported easily in water, influenced by hydrological processes including flow paths and residence times of water throughout the watershed (Cirmo and McDonnell, 1997; Band *et al.*, 2001). Collectively, nitrogen sources to landscapes along with coupled hydrological and biogeochemical processes occurring throughout the watershed strongly affect the timing and form of nitrogen delivery to surface waters and the areas of the landscape that contribute nitrogen to streams. In temperate regions, the hydrologically connected soils and land areas that drain to streams expand and contract both laterally and vertically during periods of wetting and drying. During wet periods, this causes saturated areas of the landscape to expand, especially riparian areas, which facilitates both the delivery of nitrogen to streams and its loss via denitrification. Considering such factors, environmental scientists have been successful in simulating nitrogen delivery to surface waters at many spatial and temporal scales (Creed and Band, 1998; Alexander *et al.*, 2000, 2002a; Band *et al.*, 2001; McIsaac *et al.*, 2001; Howarth *et al.*, 2002; Boyer *et al.*, 2006a).

Once nitrogen is delivered to streams or rivers, the aquatic ecosystem itself plays a critical role in modifying the nitrogen (and other material) fluxes, via channel routing and instream processing. Stream channels have a natural dendritic design that plays an intrinsic role in transporting nitrogen and other pollutants from widely dispersed upstream sources and concentrating these materials in downstream waters. Hyporheic zones of streams also play a key role in nitrogen transformations (uptake and cycling) and permanent removal (i.e., denitrification) as nitrogen is exposed to reactive benthic surfaces during transport. The hyporheic zone, literally meaning under the flow, is the zone of sediments beneath and beside the stream where surface water (from the stream) and subsurface water are exchanged, hydrologically linking this zone of sediments to the stream channel. Strong gradients in the oxygen status and nutrient content of streambed sediments occur due to hyporheic exchange, that is, the mixing of the aerated and thus well-oxygenated streamwater with deeper and anoxic subsurface flows (Bencala, 1993). Such redox gradients found in hyporheic regions create metabolically active zones that facilitate transformations of many elements of water quality. Exchange of surface water with the streambed sediments provides opportunities for denitrification to occur (Duff and Triska, 2000). Large fractions of nitrogen inputs to streams are lost via denitrification in hyporheic sediments at all scales from headwater streams to large rivers (Peterson *et al.*, 2001; Thomas *et al.*, 2001; Seitzinger *et al.*, 2002; Böhlke *et al.*, 2004; Mulholland *et al.*, 2004; Boyer *et al.*, 2006b; Triska *et al.*, this issue).

**FIGURE 1:**
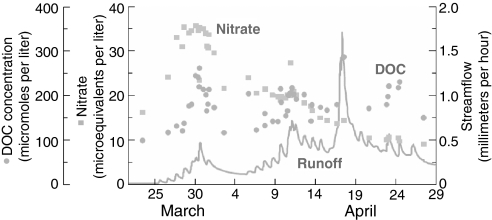
Flow Paths and Residence Times of Water in the Landscape Strongly Influence the Magnitude and Variation of Nitrate Concentrations in Headwater Streams. Reprinted from Shanley (2000).

Detailed studies of individual watersheds, where hydrological and biogeochemical processes are measured and observed over space and time, provide a scientific basis to understand the dominant factors controlling water quality and nitrogen and provide insight into how to quantify such responses at watershed and regional scales with modeling approaches. For example, the U.S. Geological Survey’s Water, Energy, and Biogeochemical Budgets (WEBB) program was designed to understand processes occurring in small watersheds located in geographically diverse environments that represent a range of hydrological, ecological, and climatic conditions. Controls on nitrogen transport and transformation over a variety of scales are being examined in nested catchments from 3 ha to 110 km^2^ (J. Shanley and S. Sebestyen, 2005, personal communication) at the Sleeper’s River WEBB site, located in the Green Mountains of northeastern Vermont. Results from this site provide a window into the importance of coupled hydrological and biogeochemical processes that affect water quality. The supply of nitrogen from this forested, headwater catchment to its receiving waters is controlled to a large degree by soil biogeochemical processes that provide sources of nitrogen from organic matter, and hydrological processes that connect the landscape to streamflow. Flow paths and residence times of water in the landscape strongly influence streamwater nitrogen concentrations. The temporal variation of nitrogen in the stream (Figure 1) is tightly linked to cycles of water (e.g., influence of spring snowmelt and associated runoff) and carbon (e.g., in dissolved organic forms, DOC), and reflects contributions of flow and solutes from both upland hillslopes and near stream riparian zones of the landscape (McGlynn *et al.*, 1999; Shanley, 2000).

**FIGURE 2:**
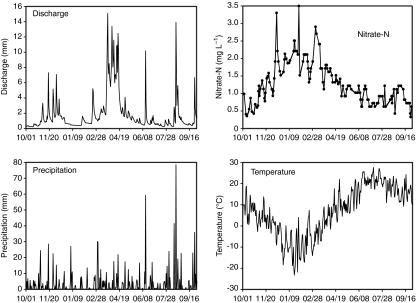
Records of Discharge, Precipitation, Nitrate-N, and Temperature at Fall Creek, NY, During 2003-04.

Such results are not limited only to small catchments, but are observed at all watershed scales. For example, nitrogen sources and fate have been studied for over 30 years in the large Fall Creek watershed in central New York, a mixed-land-use basin containing large amounts of forest (53%) and agricultural (42%) land that drain an area of 327 km^2^. Nitrogen primarily from atmospheric deposition, fertilizers, and manure, is delivered to the stream during rain and snowmelt events, with a large degree of direct connectivity of the upland landscape to the stream. Precipitation and streamflow are well distributed throughout the year (Figure 2). Despite this, instream nitrogen concentrations are notably influenced by seasonal variability, as indicated by air temperature (Figure 2). During the growing season (high temperatures), plants are able to utilize much of the nitrogen inputs to support their growth and productivity. Denitrification, a temperature-dependent process, is also important in consuming nitrogen during these periods. These results are consistent throughout the entire 30-year period of record at the site, and further illustrate the importance of coupled hydrological and biogeochemical controls affecting water quality.

### Nitrogen Transport From Headwaters to Higher-Order Streams

Mathematical models of the instream routing and biogeochemical processes that control the transport of nutrients and other solutes provide insight into the influence of headwater catchments and streams on the quality of downstream waters. The dynamics of solute transport in streams can be modeled (e.g., Stream Solute Workshop, 1990; Runkel, 1998) according to the processes of advection, dispersion, ground-water inputs, transient storage (e.g., in hyporheic zones), and nonconservative transport (e.g., uptake, denitrification). One-dimensional, steady state forms of these models provide a simplified description of nutrient transport according to a first-order exponential-decay process (e.g., Newbold *et al.*, 1981; Stream Solute Workshop, 1990; Chapra, 1997; Donner *et al.*, 2004). Nutrient transport is mediated in these models by a reaction-rate coefficient (in units of reciprocal time) and the water time of travel over a given length of stream channel (determined as the product of channel length and the reciprocal of water velocity). The steady-state reaction-rate expression reflects the aggregate, net effects of the physical, hydrological, and biochemical properties of the channel and hyporheic zone on nutrient removal. These model expressions have been advanced as part of *nutrient spiraling* concepts (Newbold *et al.*, 1981); these concepts describe the downstream transport of nutrients as a series of repeated cyclical transformations that entail nitrogen migration to the benthos via biological uptake and organic nitrogen storage and a return to the water column via mineralization and nitrification. Nutrient decay processes in these models may also include the permanent removal of nitrogen from streams via denitrification.

First-order exponential decay functions have been developed to predict nitrogen transport and losses in streams of widely varying sizes, based on empirical observations from the literature of the effects on nitrogen transport of various hydrologic and geometric properties, such as water depth, flow, velocity, and channel slope (Kelly *et al.*, 1987; Molot and Dillon, 1993; Howarth *et al.*, 1996; Alexander *et al.*, 2000, 2002a, 2004; Seitzinger *et al.*, 2002). Studies (Howarth *et al.*, 1996; Peterson *et al.*, 2001; Seitzinger *et al.*, 2002; Boyer *et al.*, 2006b) also indicate that the rates of nitrogen uptake and permanent loss via denitrification in streams generally decline in a downstream direction with increases in stream size (i.e., with increases in mean water velocity, streamflow, and depth). Headwaters and other low-order streams are important locations for nitrogen loss in river networks given that their large benthic surface area relative to the overlying water volume generally leads to greater contact and exchange of water and nitrogen with the hyporheic zone (Alexander *et al.*, 2000; Peterson *et al.*, 2001). Small streams also generally have greater benthic frictional resistance and hyporheic storage (relative to the channel water volume) than large streams and rivers (Harvey and Wagner, 2000; Harvey *et al.*, 2003), which may contribute to their higher observed rates of nitrogen loss.

Based on current understanding of these processes, land-use changes or modifications to stream channels that increase the rates of flow in headwater streams may heighten their influence on the chemical quality of downstream receiving waters. For example, increases in the peak discharge and flashiness of flows that are often associated with urbanization would be likely to reduce the natural processing of nitrogen in low-order streams, increasing the distance over which nitrogen is transported downstream. In addition, stream channelization projects that straighten channels and remove natural pools and riffles are likely to shorten the water travel time in stream reaches; this would also be likely to reduce nitrogen losses and increase downstream transport.

Some exceptions to these general patterns in nutrient transport are of note. One is the importance of floodplains and the riparian areas of large rivers, including, for example, the Mississippi and southeastern U.S. rivers, as sites for nitrogen loss via denitrification during floods. The increase in water depth during floods on these rivers actually increases the contact of nitrogen with microbially reactive floodplain sediments and promotes denitrification (NRC, 2002; Richardson *et al.*, 2004; Scott *et al.*, 2004). Another is the potential for the first-order properties of nitrogen reaction rates to break down in nutrient-enriched waters where denitrification (Garcia-Ruiz *et al.*, 1998) or uptake processes (Dodds *et al.*, 2002) become concentration saturated. Under these conditions, a lower reaction rate would be expected and nitrogen could be transported for longer distances in streams than would occur under nonsaturated conditions. Therefore, headwater catchments with high stream nitrogen concentrations, such as those found in highly urbanized or cultivated catchments, could have an even more far-reaching downstream influence than headwater streams draining relatively undeveloped catchments with low nitrogen concentrations.

Despite the extensive cycling of nitrogen and generally high rates of nitrogen loss in small streams and the terrestrial ecosystems of watersheds (e.g., Howarth *et al.*, 1996; Boyer *et al.*, 2002), there is mounting evidence that the nitrogen in downstream receiving waters is strongly connected to distant landscape sources and responds relatively rapidly to changes in these sources. These connections are observed in watershed studies at small spatial scales, such as those cited earlier, as well as in large-scale studies. One example of the latter is the Mississippi River Basin, where most of the nitrogen loadings at the Mississippi outlet to the northern Gulf of Mexico are transported from distant, inland agricultural watersheds (Alexander *et al.*, 2000). Annual changes in nitrogen load at the outlet correspond closely to contemporaneous annual changes in runoff and nitrogen inputs from agricultural fertilizers and other sources in the basin as well as changes in nitrogen inputs during the preceding 5 years (Goolsby *et al.*, 1999; McIsaac *et al.*, 2001). European studies (e.g., Stalnacke *et al.*, 2003) suggest that improvements in oxygen conditions on the northwestern shelf of the Black Sea in the early and mid-1990s near the outlet of the 800,000 km^2^ Danube River Basin occurred in response to upstream reductions in farm subsidies and the use of fertilizers in several eastern European countries following the dissolution of the former Soviet Union in 1991. The nitrogen response to fertilizer reductions has been less rapid (>10 years) in streams draining certain other eastern European watersheds (Stalnacke *et al.*, 2003).

These regional-scale studies suggest that headwater and other low-order streams may play an important role in the observed linkages between landscape pollutant sources, such as agricultural fertilizers and livestock wastes, and the long-distance transport and delivery of nitrogen to higher-order streams and coastal receiving waters. The downstream influences of landscape sources are likely facilitated by the high density of first-order (headwater) streams and their high frequency of tributary connections with all higher-order streams – properties that are intrinsic to dendritic river networks (e.g., see discussion of Tokunaga’s Law in Dodds and Rothman, 2000). These characteristics suggest that changes in the physical or chemical condition of headwaters or their catchments could potentially influence both nitrogen and flow conditions in downstream waters. In the following section, we investigate the nature of headwater connections to pollutant sources and higher-order streams and their influence on flow and nitrogen conditions in downstream waters by applying the SPARROW model to a spatially detailed network of streams and rivers.

## Assessing the downstream effects of headwaters

### Model Specification

The steady-state SPARROW model describes nutrient source inputs and one-dimensional transport in terrestrial and aquatic ecosystems, including first-order decay in streams and reservoirs. Model parameters are statistically estimated from a calibration to mean-annual nitrogen loads (mass per unit time) that are computed from periodically measured nutrient concentrations and daily flow measurements at multiple stream monitoring stations. The use of mean-annual loads in the model adjusts for temporal variability related to long-term trends and short-term changes in flow and instream nitrogen cycling and transformation processes. As a consequence, the model estimates the hydrological and biogeochemical processes that affect the long-term supply, loss, and transport of nitrogen in watersheds (Alexander *et al.*, 2000; Schwarz *et al.*, 2006). This mass-balance specification of the model is well suited for assessing the natural and human-related properties of headwaters that govern the long-term generation and transport of nitrogen and its fate in higher-order streams and downstream receiving waters. Notably, mass-balance approaches have generated considerable interest in recent years to further understanding of the long-term effects of nitrogen supply and transport on inland and coastal eutrophication (e.g., Howarth *et al.*, 1996; Vitousek *et al.*, 1997; Carpenter *et al.*, 1998; NRC, 2000; Boyer *et al.*, 2002).

The model structure, supporting equations, and details of the model estimation are given in Schwarz *et al.* (2006). Conceptually, the model is applied to individual stream reaches through a mathematical equation in which 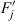 is the model-estimated mean-annual total nitrogen flux leaving reach *i*. This flux is related to the flux leaving adjacent reaches upstream of reach *i*, denoted by 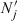 where *j* indexes the set *J*(*i*) of adjacent reaches upstream of reach *i*, plus additional flux that is generated within the incremental reach segment *i*. In most cases, the set of adjacent upstream reaches *J*(*i*) will consist of either two reaches, if reach *i* is the result of a confluence, or no reaches if reach *i* is a headwater reach. The functional relationships determining reach *i* flux are given by 
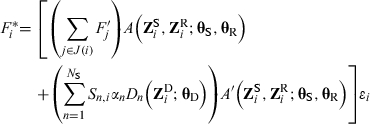
(1)

The first summation term represents the amount of flux that leaves upstream reaches and is delivered downstream to reach *i*, where *F*′_*j*_ equals measured flux, 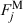, if upstream reach *j* is monitored or, if it is not, is given by the model-estimated flux 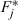. *A*(·) is the stream delivery function representing loss processes acting on flux as it travels along the reach pathway. This function defines the fraction of flux entering reach *i* at the upstream node that is delivered to the reach’s downstream node. The factor is a function of measured stream and reservoir characteristics, denoted by the vectors **Z**^S^ and **Z**^R^, with corresponding coefficient vectors **θ**_S_ and **θ**_R_. If reach *i* is a stream, then only the **Z**^S^ and **θ**_S_ terms determine the value of *A*(·); conversely, if reach *i* is a reservoir then the terms that determine *A*(·) consist of **Z**^R^ and **θ**_R_.

The second summation term represents the amount of flux introduced to the stream network at reach *i*. This term is composed of the flux originating in specific sources, indexed by *n*=1,…,*N*_S_. Associated with each source is a source variable, denoted by *S*_*n*_, and its associated source-specific coefficient, *α*_*n*_. This coefficient retains the units that convert the source variable units to flux units. The function *D*_*n*_(·) represents the land-to-water delivery factor. For sources associated with the landscape, this function, along with the source-specific coefficient, represents the rate at which the source variable is converted to nitrogen mass that is delivered to streams. The land-to-water delivery factor is a source-specific function of a vector of delivery variables, denoted by 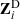, and an associated vector of coefficients **θ**_D_. For point sources that are described by a measured discharge of mass directly to the stream channel (e.g., municipal wastewater effluent), the delivery factor takes on a value of 1, with no underlying factors acting as determinants, and the estimated source-specific coefficient should be close to 1. The last term in the equation, the function *A*′(·), represents the fraction of flux originating in and delivered to reach *i* that is transported to the reach’s downstream node and is similar in form to the stream delivery factor defined in the first summation term of the equation. If reach *i* is classified as a stream (as opposed to a reservoir reach), the nitrogen introduced to the reach from its incremental drainage area receives the square root of the reach’s full instream delivery. This assumption is consistent with the notion that contaminants are introduced to the reach network at the midpoint of reach *i* and thus are subjected to only half of the reach’s time of travel. Alternatively, for reaches classified as reservoirs, we assume that the nitrogen receives the full attenuation defined for the reach.

The multiplicative error term, *ε*_*i*_, is applicable in cases where reach *i* is a monitored reach; the error is assumed to be independent and identically distributed across independent sub-basins in the intervening drainage between stream monitoring sites. Coefficient estimation is performed on the log transforms of the summed quantities in Equation (1) using nonlinear least-squares estimation (Schwarz *et al.*, 2006).

Nitrogen loss in streams is modeled according to a first-order decay process (Chapra, 1997) in which the fraction of the nitrogen mass originating from the upstream node and transported along reach *i* to its downstream node is estimated as a continuous function of the mean water time of travel (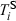; units of time) in reach *i* and a first-order reaction rate that is expressed as a power function of the mean water depth, *D*_*i*_, such that 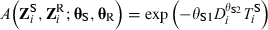
(2)where *θ*_S1_ (a coefficient in units of length^−1^ time^−1^) and *θ*_S2_ are estimated coefficients. A similar power function has been previously evaluated in SPARROW for streamflow (Alexander *et al.*, 2002a; Elliott *et al.*, 2005; Schwarz *et al.*, 2006). The nitrogen loss-rate coefficient (in units of reciprocal time), which is calculated as the product of the estimated coefficients and mean water depth, is dependent on properties of the water column that are proportional to water volume, such as streamflow and depth (Stream Solute Workshop, 1990).

Nitrogen loss in lakes and reservoirs is modeled according to a first-order process (e.g., Kelly *et al.*, 1987) in which the fraction of the nitrogen mass originating from the upstream reach node and transported through the reservoir segment of reach *i* to its downstream node is estimated as a function of the reciprocal of the areal hydraulic load 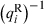 (units of length time^−1^) for the reservoir associated with reach *i* and an apparent settling velocity coefficient (*θ*_R0_; units of length time^−1^), such that 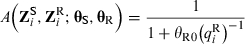
(3)

Additional details on this formulation are given in Alexander *et al.* (2002a) and Schwarz *et al.* (2006). The areal hydraulic load is estimated in this study as the quotient of the outflow discharge to the surface area of the impoundment, but may also be determined from the ratio of the mean depth to the solute residence time of the impoundment.

### Model Estimation

Our application of the model to catchments and streams in the northeastern United States is based on a previous SPARROW application (Moore *et al.*, 2004) to the 1:100,000 scale National Hydrography Dataset (NHD; USGS, 1999). The water-quality and geographic data for the nutrient sources and watershed properties are described in detail in this earlier study (Moore *et al.*, 2004). The parameters of Equations (1)–(3) are estimated using the mean-annual total nitrogen loads at 65 stream monitoring stations. The mean-annual loads were computed by applying flux-estimation procedures to daily records of flow and periodic measurements of total nitrogen concentration; total nitrogen is determined as the sum of dissolved nitrate-nitrite and total organic plus ammonia nitrogen concentration measurements (Moore *et al.*, 2004). The explanatory variables in the model include four nitrogen sources (municipal wastewater discharges, atmospheric deposition, and runoff from cultivated and developed urban and suburban lands), one terrestrial land-to-water attenuation factor (soil permeability) that is applied with equal proportional effect to all sources except municipal wastewater discharges, and a total of three nitrogen-decay coefficients for streams and reservoirs as specified in Equations (2) and (3).

The modeled region contains approximately 42,000 stream reaches having a mean catchment size of 4.4 km^2^, based on watershed boundary delineations from 30-m digital elevation data. The mean-annual streamflow for each stream reach was calculated as the sum of the mean-annual runoff for the incremental drainage area of each stream catchment and that from all upstream catchments. For 211 available gaged stream stations, most (53%) had estimated streamflows within 5% of the gaged flow; 83% had estimated flows within 10%, and 93% had estimated flows within 15% of the gaged flow. Time-of-travel estimates for Equation (2) were computed from published regression equations (Jobson, 1996) that estimate mean water velocity as a function of mean streamflow, reach slope, and the total drainage area of each stream reach. Selected properties of the approximately 23,000 headwater NHD reaches are presented in Table 1.

**TABLE 1:** Geometric and Hydraulic Properties of NHD Headwater Reaches for Northeastern U.S. Streams.

	Percentiles (Number Reaches = 23,253)				
Metric	10th	25th	50th	75th	90th
Drainage area (km^2^)	0.8	1.8	3.7	7.3	12.9
Mean-annual streamflow (m^3^/s)	0.02	0.04	0.08	0.15	0.28
Mean water depth* (m)	0.06	0.07	0.10	0.12	0.16
Mean water travel time (days)	0.02	0.05	0.09	0.14	0.19

* Depth = 0.2612*Q*^0.3966^, where *Q* is the mean-annual streamflow (Alexander *et al.*, 2000).

We estimate two additional aquatic transport functions in the model to assist in quantifying the rates of nitrogen removal in northeastern streams and lakes as a continuous function of the size and hydraulic properties of these water bodies. The parameters of these functions are estimated using current literature rates of nitrogen removal reported for streams and lakes in North America, Europe, and New Zealand (Seitzinger *et al.*, 2002; Böhlke *et al.*, 2004; Mulholland *et al.*, 2004). This information provides a generally comprehensive description of what is currently known about nitrogen transport across large spatial scales, and thus, gives a more refined method for assessing the influence of headwater sources and processes on downstream nutrient conditions.

The stream transport function describes the fraction of nitrogen mass that is transported along the experimentally studied reaches, denoted by 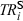 for reach *i*, expressed as a function of the stream characteristics according to 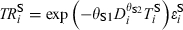
(4)where the variables and coefficients in the exponential function are identical to those in Equation (2), and 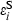 is an error term, independent across measurements, having a variance that may differ from the error term appearing in Equation (1). Literature estimates of the nitrogen transport fraction, 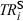, are based on denitrification and mass-balance measurements of nitrogen loss for 12 streams (see Seitzinger *et al.*, 2002; Böhlke *et al.*, 2004; Mulholland *et al.*, 2004; we use the reported estimates of the mean depth and water time of travel for the studied reaches). Many of the measurements of denitrification are based on summer, low-flow conditions and are assumed to be representative of the rates during other periods of the year.

The reservoir transport function describes the fraction of the nitrogen mass that is transported in experimentally studied lakes, denoted by 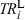 for lake *i*, expressed according to 
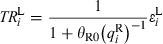
(5)where the coefficient and variable in the denominator of the expression are the same as those defined in Equation (3), and 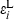 represents an independent and identically distributed error term having a variance that potentially differs from *ɛ*_*i*_ and 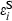 in Equations (1) and (4). The literature estimates of the nitrogen transport fraction, 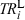, are based on denitrification and mass-balance measurements of nitrogen loss for 36 lakes (see Seitzinger *et al.*, 2002; we use the reported estimates of the mean depth and water residence time for the studied lakes to calculate the areal hydraulic load).

The three components comprising the SPARROW model consist of Equation (1) [with instream delivery fraction given by Equation (2) and reservoir delivery fraction given by Equation (3)] estimated using the instream load observations for 65 stream monitoring stations, Equation (4) estimated using the 12 literature estimates of stream delivery fraction, and Equation (5) estimated using the 36 literature estimates of lake delivery fraction. A two-step procedure was used to simultaneously estimate the coefficients of the three equations. In the first step, the model is estimated using all observations, both those associated with the monitoring station data and those associated with the literature measurements, with each observation given equal weight. The error estimates from this initial model are consistent estimates of the true errors and are used to estimate the relative variances of the three model components. The model was then re-estimated in a second step using weighted nonlinear least squares, weighting each observation according to the respective reciprocal variance (i.e., 1/RMSE^2^; RMSE = root mean square error) of the model error (weighting factors: lakes = 1/0.2925; streams = 1/0.0099; monitoring loads = 1/0.16). The weights are used to account for the level of uncertainty associated with the different types of measurements used in the model.

### Model Predictions and Simulation Methods

**FIGURE 3:**
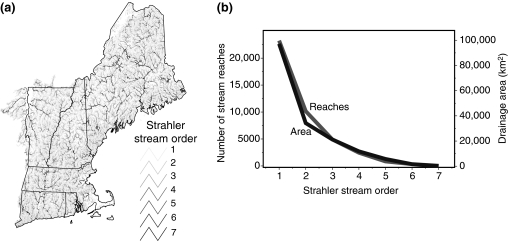
Stream Reaches From the National Hydrography Dataset for the Northeastern United States: (a) Strahler Stream-Order Number by Reach; (b) Number of Reaches and Total Drainage Area for Stream Reaches Classified by Strahler Stream-Order Number.

We use the estimated model to investigate the supply and transport of nitrogen and water in streams of varying sizes within the northeastern river network, ranging from small headwater streams to large rivers. Stream size is defined according to the Horton-Strahler stream-order number (Horton, 1945; Strahler, 1957; see Figure 3). We assigned stream-order numbers to NHD reaches using a previously developed algorithm (K. Lanfear, USGS, 2005, written communication). The Strahler ordering system produces a dendritic, hierarchical classification in which headwater streams (i.e., streams with no tributaries) are classified as order 1 with all subsequent streams of the *n*th order being located downstream of the confluence of two (*n* − 1)th order streams. The number of reaches and sum of the incremental drainage area for the NHD streams both decline at a similar rate with increasing stream order (see Figure 3b) that is generally consistent with Horton’s geometric-scaling laws. These scale-invariant laws correspond to the fractal structure of drainage networks (Peckham and Gupta, 1999) and describe fundamental mathematical properties that relate to the similar spatial organization of various topographic and geometric properties, including stream number, drainage area, and stream length, throughout the hierarchy of stream network systems (Rodriguez-Iturbe and Rinaldo, 1997; Peckham and Gupta, 1999).

We use the Horton-Strahler stream classification with the model predictions to quantify the pollutant sources and rates of nitrogen delivery within streams of varying sizes in the northeastern NHD river network. We track nitrogen delivery to NHD reaches from the four pollutant sources within the *incremental* drainage area of each reach. The *incremental* area of a stream reach is defined as the catchment drainage area from which water and nitrogen directly enter the reach, independent of the drainage area of upstream reaches that hydrologically connect to the reach. We summed the mass of nitrogen delivered from all incremental drainage areas of NHD stream reaches within each Strahler stream-order class and for each pollutant source. Similarly, we also use the network data on streamflow to quantify the flow contributions from the incremental drainage areas of different sized NHD reaches by summing the incremental reach flows separately among reaches with similar Strahler stream-order numbers.

We use several model simulations to investigate the influence of nitrogen sources, streamflow, and instream processing in headwater catchments on the mean-annual nitrogen and flow conditions in downstream waters. First, to quantify the downstream contributions of headwater nitrogen loads, we set the total inputs from all nitrogen sources in headwater streams to zero in the model and track the resulting change in nitrogen loads in all higher-order streams (orders 2-7). The results quantify the percentage of the downstream loads in each Strahler stream-order class that originates collectively from the 23,253 headwater catchments. Similar evaluations for mean-annual flow quantify the percentage of the flow in each stream-order class that originates from headwater catchments.

Second, we refine the model simulations to investigate the downstream effects on nitrogen loads from changes in pollutant sources in various collections of randomly selected headwater catchments. These simulations, which randomly select from 10% (2,325 reaches) to 90% of the reaches (20,928), give useful information about the sensitivity of the downstream changes in loads when significant changes occur in the pollutant sources in a subset of headwater reaches.

Finally, to quantify the downstream effects of loss processes (e.g., denitrification) in headwater streams and reservoirs, we set the decay rate to zero in headwater streams and reservoirs and track the change in the nitrogen loads in first- and all higher-order streams. For each stream-order class, we compute the mean of the percentage changes and the standard deviation among all reaches, with the latter metric indicating the spatial variability among streams of the same order. The adjustment to the decay rate in these simulations is identical to setting the water travel time (or areal hydraulic load for reservoirs) to zero because both impart identical effects in the decay functions given in Equations (2) and (3).

### Results of the Model Estimation

The parameter coefficients and model performance statistics are given in Table 2. The model explains 95% of the spatial variability in log-transformed mean-annual total nitrogen loads (i.e., *R*^2^ = 0.95). All model coefficients are statistically significant for *α* = 0.10. The prediction accuracy is ±44% for individual reaches, based on the RMSE of the model for one standard deviation variability. Model predictions of nitrogen yields from predominantly forested, cultivated, and developed urban and suburban catchments compare favorably with those reported in the literature for similar land uses (e.g., Beaulac and Reckhow, 1982). For example, predicted yields from forested catchments (median = 2.7 kg/ha/year; interquartile range from 1.8 to 3.4 kg/ha/year) are 20-25% of the predicted yields for cultivated and developed catchments.

**TABLE 2:** Estimated Coefficients for the SPARROW Total Nitrogen Models for Northeastern U.S. NHD Streams.

	Estimated model*		
Predictor Variables	Coefficient	Units	Standard Eror
Sources			
Municipal wastewater	1.42	Dimensionless	0.39
Atmospheric deposition	0.412	Dimensionless	0.058
Cultivated agricultural land	678	kg/km^2^/year	260
Developed urban and suburban land	726	kg/km^2^/year	232
Land-to-water delivery			
Soil permeability	0.387	Dimensionless	0.154
Instream loss			
θ_S1_	0.0513	m^−1^ day^−1^	0.0084
θ_S2_	-1.319	dimensionless	0.076
Reservoir/lake loss	9.9	m/year	1.6
Number of observations	113		
*R*^2^	0.95		
RMSE (root mean square error in %)	44.2		

* The model as defined by Equations (1)-(5) is estimated using load data for the 65 stream monitoring sites and additional literature measurements of the nitrogen loss rate in streams (*N* = 12) and lakes (*N* = 36) in New Zealand, North America, and Europe (data are from Seitzinger *et al.*, 2002; Böhlke *et al.*, 2004;Mulholland *et al.*, 2004).

**FIGURE 4:**
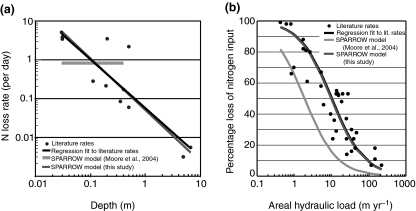
Nitrogen Loss in Streams, Lakes, and Reservoirs: (a) Streams in Relation to Mean Water Depth and (b) Reservoirs and Lakes in Relation to the Areal Hydraulic Load. The literature rates are for streams and lakes in North America, Europe, and New Zealand (Seitzinger *et al.*, 2002; Böhlke *et al.*, 2004; Mulholland *et al.*, 2004). The literature rates in (a) were originally reported as a percentage of nitrogen inputs in Seitzinger *et al.* (2002) and are converted to first-order rates here using the corresponding measurements of the water time-of-travel. The regression fit for the literature rates in (a) is obtained from a log-linear regression: *k* = 0.0573*d*^−1.246^, where *k* is the first-order rate coefficient and *d* is the mean water depth; *R*^2^ = 0.770. The regression fit for the literature rates for lakes in (b) is obtained from a nonlinear regression: *N* = 1 − [1/(1 + 10.4*q*^−1^)], where *N* is the fractional nitrogen loss and *q* is the areal hydraulic load; *R*^2^ = 0.757; the estimates are virtually identical to those estimated in the SPARROW model in this study.

The inclusion of literature nitrogen loss rates in the model estimation provides sufficient statistical power to quantify nitrogen loss as a continuous function of the hydraulic conditions in streams and reservoirs in the northeastern United States (Table 2; Figure 4). We find that the continuous stream loss function gives first-order nitrogen loss rates (Figure 4a) that decline with increases in mean water depth (also mean streamflow). This inverse relation is consistent with that reported for other SPARROW nitrogen models (Alexander *et al.*, 2002a; Schwarz *et al.*, 2006) and is also consistent with the widely held scientific notion that water-column nitrogen loss rates generally decline with increasing water depth (e.g., Stream Solute Workshop, 1990; Peterson *et al.*, 2001; Thomas *et al.*, 2001). The rates estimated here for small streams (depths < 0.39 m) are generally consistent with the single loss rate (0.82 day^−1^) that was estimated according to a discrete loss function in the previous northeastern SPARROW model (Moore *et al.*, 2004). The first-order rates from the continuous loss function (Figure 4a) are centered on the previously estimated constant rate and provide a reasonable description of the dimensions of the inverse relation over these smaller stream sizes. Although the literature data include relatively few observations of nitrogen loss in larger streams (those with depths greater then 0.39 m; Figure 4a), these observations provide important complementary information for estimating nitrogen losses in streams of the Northeast. Attempts to estimate the model with a continuous instream loss function (i.e., Equation (2)) using only the load data from the 65 monitoring sites were unsuccessful as the model failed to converge.

The estimated nitrogen loss coefficient (i.e., mass-transfer rate) for reservoirs (Table 2) is similar to that estimated for the lake data alone (Figure 4b) – i.e., 9.9 m/year compared with 10.4 m/year, respectively – and is about five times larger than that estimated in the previous northeastern SPARROW model (Moore *et al.*, 2004; i.e., 9.9 m/year compared with 1.9 m/year, respectively). Based on a re-estimation of the coefficients in this previous model using a fixed reservoir mass-transfer coefficient value of 9.9 m/year, we find that a difference in the reservoir loss rate coefficient of this magnitude has relatively little effect on the estimates of the other coefficients in the earlier model. The general insensitivity of the model coefficients to such changes is consistent with suggestions by Moore *et al.* (2004) that the monitoring sites may be poorly located in relation to the reservoirs in the northeastern catchments. However, relatively small rates of nitrogen loss in reservoirs are generally consistent with previous SPARROW models applied in the United States (Smith *et al.*, 1997) and New Zealand (Alexander *et al.*, 2002a).

Other comparisons with the previous northeastern model (Moore *et al.*, 2004) indicate that the model estimated here gives an equally plausible description of nitrogen sources and transport in the northeastern catchments and streams. Although the estimated model yields a slightly higher model error (RMSE = 44.2%) as compared with that for the previous model (RMSE = 40.4%), the changes in the mean estimates of the model coefficients are within the measures of uncertainty as expressed by the standard errors of the coefficients. Differences in the quantities of nitrogen delivered to streams from the various sources are relatively small; the model reported here (Table 2) indicates that the contributions from municipal wastewater sources are about 25% higher than estimated in the previous model, whereas the nitrogen contributions from cultivated and developed urban/suburban lands are about 25% lower. Predictions of nitrogen yield for about 6,600 catchments with predominantly cultivated, developed urban/suburban, or forested land uses differ by less than 25% from the model predictions generated by the previous model.

### The Supply and Delivery of Nitrogen and Water to Streams

**FIGURE 5:**
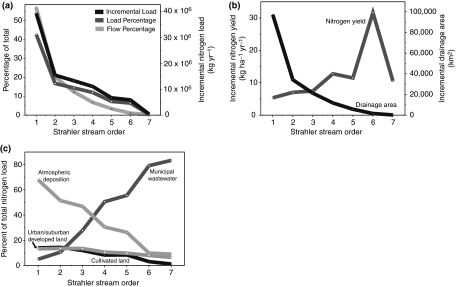
Mean-Annual Streamflow and Total Nitrogen Loads, Yields, and Sources for Streams of the Northeastern United States in Relation to Strahler Stream-Order Class: (a) Load From the Incremental Reach Watersheds and the Load and Flow, Expressed as a Percentage of the Sum of the Incremental Load and Flow in Streams of All Orders; (b) Yield and Drainage Area of the Incremental Reach Watersheds; (c) Sources of the Nitrogen Loads, Expressed as a Percentage of the Sum of the Incremental Load in Streams of the Same Order.

Based on comparisons of model predictions of flow and the nitrogen loads for the incremental drainages of NHD streams of varying sizes (as defined by Horton-Strahler class; Figure 5), headwaters catchments, in aggregate, account for nearly one-half of the total nitrogen mass supplied to all streams – i.e., headwaters account for 45% of the total nitrogen mass or load that is delivered to all stream reaches from the incremental drainage areas of reaches in the northeastern NHD river network (Figure 5a). By comparison, second- and higher-order streams account for less than 20% of the total nitrogen load that is delivered to all streams. This percentage declines progressively (as does the drainage area; Figure 5b) with increases in stream order.

The nitrogen yields (i.e., loads per unit drainage area) from the incremental drainages (Figure 5b) of headwater streams (mean = 5.5 kg/ha/year) are among the smallest among all stream orders. Atmospheric deposition is the largest source of nitrogen in headwater catchments, accounting for nearly 70% of the total incremental load delivered to headwater streams, with cultivated land and urban/suburban sources accounting for about 27% of the incremental load (see Figure 5c). Most headwater catchments where atmospheric deposition is high are predominantly forested; more than 50% of the headwater catchments have more than 85% forested land area. Cultivated and urban/suburban lands account for more than 10% of the land area in about 75% of the headwater streams. The nitrogen yields increase progressively with stream order (Figure 5b), reflecting the increase in municipal wastewater discharges associated with increases in population in the vicinity of the higher-order streams (see Figure 5c). The large increase in yield in stream order 6 (Figure 5b) includes incoming loads to the lower Connecticut River, where major municipal wastewater discharges occur; note that the percentage of the total incremental load attributable to wastewater discharges increases from 50% in stream order 5 to nearly 80% in stream order 6. Overall, these results indicate that, although the nitrogen yields in headwater streams are generally the smallest among all stream orders (Figure 5b), collectively, the total loads of nitrogen leaving headwater reaches are similar in size to the sum of all loads that originate in the incremental watersheds of higher-order streams.

The mean-annual flow contributions from the incremental drainage areas of NHD reaches (Figure 5a) indicate that first-order streams account for approximately 60% of the total volume of mean-annual flow that is contributed to all northeastern streams. Similar to that observed for other stream properties (e.g., nitrogen load, drainage area), the flow contributions that originate in the incremental watersheds of higher-order streams, expressed as a percentage of the total flow volume in all streams, are relatively small and decline monotonically with increases in stream order, from about 20% for second-order streams to less than 1% for sixth- and seventh-order streams.

### Downstream Influences of Headwaters

**FIGURE 6:**
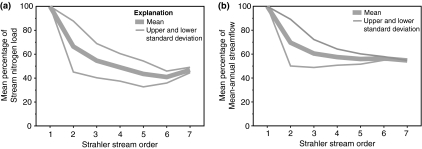
The Percentage of the Mean-Annual Nitrogen Load and Streamflow in Streams of the Northeastern United States That Originates in Headwater Catchments: (a) Nitrogen; (b) Streamflow. The estimates are obtained in model simulations by setting the total nitrogen source loadings or streamflow to zero in 23,253 headwater (Strahler order 1) catchments and quantifying the resulting percentage change in the downstream nitrogen loads or flow. The upper and lower standard deviation lines reflect the range of variability (associated with one standard deviation) observed in reaches in the estimated percentage reduction in nitrogen load.

**FIGURE 7:**
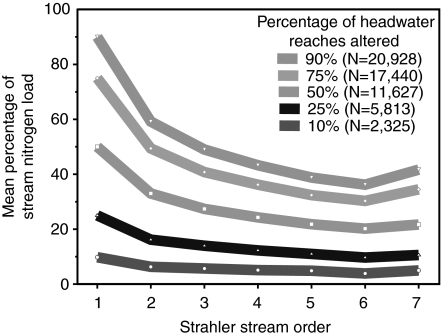
The Mean Percentage of the Stream Nitrogen Load in National Hydrography Dataset Reaches Originating From Randomly Selected Subsets of the Headwater Catchments in Relation to Strahler Stream-Order Class. The estimates are obtained in model simulations by setting the total nitrogen source loadings to zero in a randomly selected set of headwater catchments, ranging in number from 2,325 (10% of the total reaches of 23,253) to 20,928 reaches (90% of the total), and quantifying the resulting percentage change in the downstream nitrogen loads.

The results of the model simulations (Figures 6 and 7) indicate a demonstrable effect of the nitrogen sources and flow in headwater catchments on the mean-annual nitrogen and flow conditions in downstream reaches. The percentage of the mean-annual nitrogen load in reaches that is contributed from headwater streams steadily declines with increases in stream order through the sixth-order streams (Figure 6a). We found that second-order streams receive approximately 65% of their nitrogen loads from headwater streams. This percentage contribution of headwater streams ranges from 43% to 87% of the nitrogen loads in second-order streams, based on the two-thirds of the streams that lie within a one standard deviation range in this stream-size class. The lowest contribution of headwater streams to nitrogen loads is about 40% as observed in sixth-order streams. The higher fraction of headwater nitrogen contributions in streams of order 7 as compared with order 6 reflect differences in the load response and potentially the network structure of two independent river basins, the Connecticut and Penobscot (we executed separate simulations for these drainages and found monotonically decreasing headwater contributions with increasing stream order in each basin that are similar to those shown in Figure 6a for stream orders 1-6).

We find that the percentage of the mean-annual flow in network streams that originates from headwater catchments exhibits a monotonic decline from headwaters to high-order streams similar to that found for nitrogen loads, but is somewhat larger in magnitude than observed for the nitrogen loads (Figure 6b). Headwater catchments contribute approximately 70% of the water volume in second-order streams. Moreover, the flow contributions of headwater catchments to the mean water volume in downstream reaches decline only marginally to about 55% in fourth- and higher-order streams.

The large contributions of headwater nitrogen sources and flow volumes to mean-annual nitrogen loads and flow in streams of all sizes are generally consistent with the high density of headwater streams and the high frequency of their connections to the channels of all higher-order streams; these are intrinsic properties of dendritic river networks. The proportion of all lower-order streams that are tributary to streams of a given Strahler order conforms to fundamental scaling properties defined according to Tokunaga’s Law (e.g., see discussion in Dodds and Rothman, 2000). According to this law for commonly observed values of network scaling parameters (Tokunaga, 2003), first-order streams represent the single, most prevalent Horton-Strahler stream-order class with high frequencies of tributary connections to all higher-order streams within river networks. Considering all of the lower-order tributaries to higher-order streams in a network, the percentage of lower-order streams that are theoretically classified as first-order declines with an increase in stream order, but levels off to about 50% (see Table 3). These percentages of first-order tributary connections to higher-order streams are generally similar for the northeastern NHD river network. Therefore, first-order streams are the most frequently occurring tributary to all higher-order streams and represent the origin of a major fraction of the water and nitrogen loadings in streams of all sizes within the northeastern United States.

**TABLE 3:** Headwater Tributary Connections to Higher-Order Streams in River Networks.

	Headwater (First-Order) Streams		
	Percentage of All Lower-Order Tributary Reaches Classified as First-Order Streams		
Strahler Stream-Order Class	Theoretical*	New England NHD	Number of NHD Stream Reaches
2	100.0	100.0	11,775
3	66.7	46.5	5,019
4	57.1	54.3	2,527
5	53.3	57.7	1,181
6	51.6	53.5	497
7	50.8	51.1	45

* The estimates are based on Tokunaga’s law for describing the average number of streams of a given order that are tributaries to higher-order streams (Dodds and Rothman, 2000). For common values of the network scaling parameters (Tokunaga, 2003), the average number of first-order tributaries to higher-order streams of order *v* is computed as 2^*v*−1^. In the table, the average number of first-order tributaries to a specified stream order is expressed as a percentage of the total number of all lower-order connecting tributaries for that stream order.

Refinements to the model simulations to assess the downstream effects of changes in nitrogen sources in a subset of the headwater catchments (Figure 7) provide insight into the magnitude of the water-quality effects in cases where pollutant sources and land use undergo significant changes in a subset of headwater streams. We find that the mean percentage of the stream nitrogen load that originates in headwater catchments declines monotonically with increases in Strahler stream order through the sixth-order streams; the mean percentage shows an approximate leveling in magnitude in fourth- and higher-order streams. The rate of decline is generally similar for simulations involving changes in sources in 50% or more of the headwater reaches; a slightly smaller rate of decline is noted in the mean percentage for simulations involving fewer headwater reaches. The results indicate that nitrogen sources in as few as 50% of the headwater catchments account for 20-25% of the nitrogen loadings in fourth- and higher-order streams; sources in as few as 25% of the headwater catchments account for 10-12% of the nitrogen loadings in fourth- and higher-order streams.

A simulation of the downstream effects of nitrogen loss processes in headwater streams and reservoirs (related to denitrification and long-term storage) indicates that nitrogen losses in headwaters reduce the nitrogen loads by about 8% in first-order (headwater) streams (standard deviation = ∼0-30%), 5% in second-order streams (standard deviation = <1-12%), and about 3-4% in fourth- and higher-order streams. These estimates are calculated as the change in simulated load expressed as a percentage of the original decayed load. The reported changes in load reflect the integrated effects of instream biochemical processing (e.g., denitrification) and water travel times within stream reaches (see Table 1) on the rates of stream nitrogen loss (note that the nitrogen delivered to headwater stream channels from point- or land-based sources is assumed to enter, on average, the midpoint of total channel length of the headwater reach and is therefore subjected to only half of the water time of travel). The large variability in nitrogen loss in headwater streams (i.e., ∼0-30%) reflects differences among first-order reaches in the mean water depth and water travel time. Although nitrogen losses in headwaters streams cause relatively small changes in the nitrogen loads in higher-order streams on average, the downstream change in nitrogen loads is actually large relative to the change in headwater loads – i.e., the downstream relative changes in load range from 40% to 60% of the relative change observed in the headwater nitrogen loads.

### Uncertainties and Research Needs

Headwater streams are operationally defined in our assessment as Horton-Strahler first-order perennial streams, based on the 1:100,000-scale NHD river network. The Horton-Strahler classification of NHD streams gives a reasonable approximation of headwater locations in relation to those of higher-order streams within the larger drainage network. This definition is based on fundamental principles that describe the hierarchy of the spatial organization of various topographic, hydrologic, and geometric properties of river networks. Comparisons of the Horton-Strahler classification of NHD streams with classifications for more finely resolved 1:24,000-scale streams (Andrews *et al.*, 2002) suggest that NHD headwater channels may be generally classified as second-order streams at this finer scale. Thus, the first-order headwater streams in our study reflect the flow and nitrogen contributions from many smaller streams, including those from intermittent ephemeral streams.

The use of the Horton-Strahler classification to define headwaters has received some criticism (e.g., Gomi *et al.*, 2002; Whiting and Bradley, 1993) because it does not explicitly include hydrological and biological process-related definitions of transitional upland headwater reaches; these are reach locations where the influence of hillslope processes on water and material flux tends to give way to the fluvial routing processes that dominate in higher-order streams. There are, however, intrinsic ambiguities in defining headwater streams that arise from the dynamic spatial and temporal nature of hydrological and biological processes in low-order streams; this contributes, for example, to the lack of consistent definitions of intermittent and ephemeral headwater streams (Meyer and Wallace, 2001).

Additional studies are needed to investigate the effects on our interpretations of alternative definitions of headwater streams in relation to various hydrological- and biogeochemical-process characteristics. This research will demand the use of more spatially detailed digital topography (e.g., 1:24,000 or finer scales) as well as equally refined watershed data, including data on climatic conditions, point and diffuse contaminant sources, and instream nutrient concentrations, for use as input to regional-scale source-transport models.

Our model analyses assume that mean-annual, instream nitrogen losses can be described as a first-order process, mediated by a loss-rate coefficient, the mean-annual solute travel time within stream channels, and mean water depth (or mean-annual streamflow). The first-order assumption of the loss process is potentially subject to some uncertainties, related to the limiting effects of saturation kinetics on denitrification rates (e.g., Garcia-Ruiz *et al.*, 1998), especially in highly developed watersheds where high nitrate concentrations can occur. Under such conditions, for example, highly developed headwater catchments could have more far reaching downstream effects than under the assumed first-order kinetics of the model. The first-order loss function also reflects the aggregate, net time-averaged effect of the hydraulic and biogeochemical properties of streams of varying size; this function does not isolate the effects of specific properties of the benthic sediment, such as organic carbon and oxygen content.

Although our modeling analysis is well suited to examine the natural and human-related processes that control the downstream transport and fate of the nitrogen over annual or longer time periods, it does not include any explicit assessment of the effects of seasonal or other temporal variability in nitrogen loss and streamflow (e.g., heterotrophic and autotrophic production and respiration) on the transport and downstream fate of nitrogen. These short-term processes are included in dynamic mechanistic models (e.g., HSPF; Bicknell *et al.*, 2001), but these models are rarely used to track the geography of nitrogen losses and the downstream transport and fate of nutrients in large watersheds (e.g., Filoso *et al.*, 2004). One difficulty is that the influence of short-term uptake and cycling processes on the downstream fate of various nitrogen forms is not currently well understood, based on available experimental research (Peterson *et al.*, 2001; Grimm *et al.*, 2003). Considerable progress has been made in measuring nitrogen cycling at the reach and catchment scales in small streams (e.g., Peterson *et al.*, 2001; Hall and Tank, 2003; Mulholland *et al.*, 2004; Royer *et al.*, 2004), but longitudinal studies are needed to quantify the effects of autotrophic and heterotrophic uptake and cycling of nutrients in low-order streams on nutrient conditions in higher-order systems. This includes an improved tracking of the separate fate of organic and inorganic nitrogen in models to enhance understanding of the headwater origins of bio-available nitrogen in downstream waters. Observational data and model improvements are also needed to account for the effects of long ground-water residence times that can delay the delivery of nitrogen from land-based sources to downstream waters (e.g., Böhlke and Denver, 1995; McIsaac *et al.*, 2001).

## Conclusions

Our synthesis of existing watershed research and the modeling assessment of northeastern U.S. streams demonstrate the important role that headwaters play in the supply, transport, and fate of water and nitrogen in river networks. This provides important information for the water-resource community regarding decisions on the regulation and management of headwater streams. The results also provide scientific information that potentially broadens understanding of the extent of Federal CWA jurisdiction in waters of the United States, a topic of continuing importance as indicated by recent U.S. Supreme Court cases. The procedures for establishing Federal jurisdiction that have emerged from these cases stress the need for technical and scientific information about whether a “significant nexus” exists between upland waters and downstream navigable waters and their tributaries. Such a connection could be based on evidence that the use, degradation, or destruction of non-navigable headwaters demonstrably influences the waters covered by the CWA.

The results reported here are consistent with the notion that pollutant sources and hydrological and biogeochemical processes in headwaters are physically and bio-chemically connected to the water-quality conditions in downstream waters of widely varying sizes, including navigable waters and their tributaries. Experimental studies of nitrogen transport in streams and rivers indicate that hydrological processes in headwater catchments influence stream nitrogen conditions by controlling the recharge of subsurface water stores and the flow paths and residence times of water through landscapes. The dynamic coupling of hydrological and biogeochemical processes in upland streams further controls the chemical form, timing, and longitudinal distances of nitrogen and other solute transport to downstream waters. Headwater influences on water-quality conditions in downstream waters are likely facilitated by the high density of headwater streams and their high frequency of tributary linkages to the channels of higher-order streams in river networks. These natural dendritic properties of stream networks play an intrinsic role in the delivery of nitrogen and other pollutants to downstream receiving waters from headwater locations throughout watersheds.

Our application of a refined version of the source-transport model SPARROW illustrates many of these concepts. The results demonstrate the prominent influence of headwaters on the mean-annual flow and nitrogen conditions in streams of all sizes in the northeastern United States. We estimate that headwater catchments contribute a majority (∼65%) of the nitrogen mass and water volume (∼70%) in second-order streams; these contributions decline only marginally to about 40% and 55%, respectively, in fourth- and higher-order streams. We also find that the downstream effects of headwater pollutant sources of nitrogen are generally very large in absolute terms in comparison to the effects of instream processing and long-term nitrogen storage in headwater streams. Nevertheless, the downstream effects of nitrogen processing and storage within headwater streams are still quite large in relative terms, ranging from about 40% to 60% of the magnitude of the relative effects observed in the headwater reaches. Moreover, because of the larger magnitude of nitrogen loads in downstream waters, the magnitude of the change in loadings related to headwater processes is actually quite large in absolute units of nitrogen mass. Our assessment of the potential downstream effects on nitrogen loads related to significant changes in land use or flows in headwater catchments indicates that the downstream nutrient loads change by approximately 50% of magnitude of the percentage of headwater reaches in which these changes occur. Thus, for example, major changes in nitrogen loads in a subset of 25% of the headwater catchments would be expected to change nitrogen loads by about 10-12% in the waters downstream of these headwaters. In view of the comparatively larger headwater flow contributions to downstream waters, we would anticipate generally larger downstream effects on mean-annual streamflow in response to major changes in the land use (e.g., pervious cover) or channel properties (e.g., channelization, water velocity) in headwater catchments and streams.
